# Bacteria Adhesion of Textiles Influenced by Wettability and Pore Characteristics of Fibrous Substrates

**DOI:** 10.3390/polym13020223

**Published:** 2021-01-11

**Authors:** Tahmineh Hemmatian, Halim Lee, Jooyoun Kim

**Affiliations:** 1 Department of Textiles, Merchandising and Fashion Design, Seoul National University, Seoul 08826, Korea; hanh11@snu.ac.kr (T.H.); hlee335@snu.ac.kr (H.L.); 2 Research Institute of Human Ecology, Seoul National University, Seoul 08826, Korea

**Keywords:** bacteria, adhesion, polystyrene, poly(lactic acid), electrospun web, wetting, morphology, pore, plasma treatment, *Staphylococcus aureus*, *Escherichia coli*

## Abstract

Bacteria adhesion on the surface is an initial step to create biofouling, which may lead to a severe infection of living organisms and humans. This study is concerned with investigating the textile properties including wettability, porosity, total pore volume, and pore size in association with bacteria adhesion. As model bacteria, Gram-negative, rod-shaped *Escherichia coli* and the Gram-positive, spherical-shaped *Staphylococcus aureus* were used to analyze the adhesion tendency. Electrospun webs made from polystyrene and poly(lactic acid) were used as substrates, with modification of wettability by the plasma process using either O_2_ or C_4_F_8_ gas. The pore and morphological characteristics of fibrous webs were analyzed by the capillary flow porometer and scanning electron microscopy. The substrate’s wettability appeared to be the primary factor influencing the cell adhesion, where the hydrophilic surface resulted in considerably higher adhesion. The pore volume and the pore size, rather than the porosity itself, were other important factors affecting the bacteria adherence and retention. In addition, the compact spatial distribution of fibers limited the cell intrusion into the pores, reducing the total amount of adherence. Thus, superhydrophobic textiles with the reduced total pore volume and smaller pore size would circumvent the adhesion. The findings of this study provide informative discussion on the characteristics of fibrous webs affecting the bacteria adhesion, which can be used as a fundamental design guide of anti-biofouling textiles.

## 1. Introduction

Bacteria adhesion and growth on materials are of great concern in many circumstances for public health and safety. In particular, textiles that we constantly contact can be exposed to numerous types of bacteria and can act as a media to deliver bacteria and spread infectious diseases [1,2,3]. Since the textiles have both roughened surfaces and pores within their volume, they provide a dynamic environment for bacteria to adhere, grow, and form biofilms. Such being the case, researchers were motivated to develop antibacterial textiles that can control bacteria adhesion and growth [4,5,6,7,8]. For example, as a preventative measure to bacterial growth on textiles, various antimicrobial treatments such as quaternary ammonium compounds, triclosan, and chitosan have been incorporated into fibers [9]; however, those materials often fail to kill every organism effectively. Biocidal nanoparticles including silver [10,11,12], copper [12,13], titanium dioxide [14,15,16], and zinc oxide [17,18,19] are also used, where silver is particularly effective at interfering with bacterial metabolism. However, the adverse effect of those reactive species on human cells is still obscure, and the environmental and toxicological [20,21,22] concerns about using nanoparticles still remain. Moreover, the abusive use of antibiotics as antimicrobial treatment lead to antibiotic resistance of cells, aggravating the associated risks. Thus, non-maleficent treatment is highly needed but still challenging [23].

As an alternative approach, controlling the bacteria adhesion by modifying the physical properties of textiles has been largely explored [24]. To this end, multiple physical aspects of materials have been considered, including surface energy, wettability, surface charge, and topography. Among the parameters, wetting has been considered as the most relevant parameter dictating the microbial adhesion on surfaces [25]. Previous studies examined the effect of wettability on bacteria adhesion, while the results are hardly conclusive [26]. Superhydrophobic surfaces with a water contact angle >150° with a low roll-off angle have been generally reported to be anti-adhesive to bacteria cells by weakening the bacterial adhesion. This phenomenon is called as “self-cleaning ability”, where loosely adhered bacteria are removed easily by gentle rinsing. Moderate hydrophobic surfaces often showed immense adherence of bacteria, especially for the Gram-negative cells with lipopolysaccharide membrane [27]. Bacteria adhesion on a hydrophilic surface is argumentative; some studies showed an intensified adhesion of cells on hydrophilic surfaces, regardless of Gram characteristics of cells, and some other studies reported the anti-adhesive properties against cells for hydrophilic surfaces [20,27,28,29]. 

The effect of the spatial distribution of roughness patterns on bacteria adhesion has been reported [30]. However, how nano- and microscale roughness patterns in textile influence bacteria adhesion could not be generalized, since bacteria’s size and shape also play important roles in interactions with material surfaces. The complexity also comes from the fact that the roughness, together with surface energy, affects the wetting property [31,32,33]. When roughness is introduced onto a low surface energy material, the surface’s wettability is further reduced, ultimately leading to a superhydrophobic surface. Yet, additional studies are needed on the spatial distribution of roughness structures and macroscopic/microscopic patterns on fibrous surfaces relative to bacterial size to determine the roughness criteria of geometries and scales that most effectively control the bacteria adhesion. A material’s surface property defines a considerable part of cell–surface interaction [34,35]. Although important, the surface property itself may not fully explain the interaction between the microbes and the materials. Textile material retains numerous pores in its volume, and those pores may act as trap sites for bacteria. While it may be essential to consider the pore characteristics and roughness of textile materials in assessing the bacteria adhesion, those factors rarely have been investigated.

The specific aim of this study is to identify textile properties that control bacteria adhesion by investigating the effect of textile material’s wettability, morphology, porosity percentage, total pore volume, and pore size. In consideration of an application to disposable hygiene products made of nonwovens, electrospun webs made from polylactic acid (PLA) and polystyrene (PS) were used in this study, with modifications of surface chemistry via O_2_ plasma and plasma-enhanced chemical vapor deposition (PECVD) of C_4_F_8_. PS and PLA were chosen for being hydrophobic and readily processable for electrospinning. The plasma treatment method, as a dry process, was employed for surface modification, to avoid the toxic wet process. As model bacteria, Gram-negative, rod-shaped *Escherichia coli* (*E. coli*) and Gram-positive, round shape *Staphylococcus aureus* (*S. aureus*) strains were employed. This study is novel in that pore characteristics, which are unique to textile materials, were considered in interpreting the interaction between bacteria cells and textile materials. Most earlier studies were focused on the surface roughness patterns and the wetting properties concerning bacteria adhesion, and little has been discussed on the effect of pore characteristics. In this study, the cell adhesion and the retention inside the material volume were considered in explaining the bacteria adhesion on textiles; this approach is not only novel but also realistic for textile applications. By understanding the fiber material characteristics affecting the bacteria adhesion, a proper design of textiles could be suggested to circumvent the adherence of infectious bacteria. The results of this study would ultimately contribute to enhancing the hygiene aspect of textiles and to reducing the malfunction attributable to bacteria adhesion.

## 2. Materials and Methods

### 2.1. Materials

Gram-negative strain *Escherichia coli* KCTC 1039 (*E. coli*) and Gram-positive strain *Staphylococcus aureus* ATCC 6358 (*S. aureus*) were used as model bacteria. Polylactic acid (PLA) resin (Ingeo 4043D, 98% L-lactide, Mw~111,000) was obtained from NatureWorks (Green Chemical Co., Ltd., Seosan-si, Chungcheongnam-do, Korea). Polystyrene (PS) pellets (Mw ≈ 350,000), tetrahydrofuran (THF), dimethylformamide (DMF), dichloromethane (DCM), dimethyl sulfoxide (DMSO), chloroform, toluene, and methylene iodide were purchased from Sigma-Aldrich (St. Louis, MO, USA). Phosphate-buffered saline (PBS, pH 7.4) was purchased from Thermo Fisher Scientific (Fisher Scientific Korea Ltd., Incheon, Korea). Iodonitrotetrazolium chloride (INT) was purchased from TCI Chemicals (Tokyo Chemical Industry Co., Ltd., Tokyo, Japan), and culture Luria–Bertani broth (LB) was purchased from ATS Korea (Yongin-si, Gyeonggi-do, Korea).

### 2.2. Film and Electrospun Web Preparation

The polylactic acid film (PLA-film) and polystyrene film (PS-film) were prepared by spin-coating (MIDAS spin coater, SPIN-1200D, Daejeon, Korea) 3 mL of 15 % (*w/v*) PLA in chloroform and 20 % (*w/v*) PS in a 1:1 volume ratio of chloroform and toluene. The spinning speed was 500 rpm, and the spinning time was 35 s. PLA and PS fibrous webs were prepared by electrospinning (ESR200PR2D, NanoNC, Seoul, Korea). A 10% (*w/v*) PLA solution with a 1:1 volume ratio of DCM and DMF was electrospun at a feeding rate of 3 mL/h at 10 kV. The needle gauge of 22 was used, and the tip-to-collector distance was 10 cm. For the PS fiber web, 18% (*w/v*) PS solution and 25% (*w/v*) PS solution were prepared, respectively, in a 1:1 volume ratio of THF and DMF. An 18% PS solution was electrospun at the feeding rate of 2 mL/h and voltage of 22 kV with 12 cm of tip-to-collector distance. A 25% PS solution was electrospun at the feeding rate of 2 mL/h and voltage of 13 kV, with the tip-to-collector distance of 15 cm. For both PS electrospinning, a 23-gauge needle was used.

### 2.3. Surface Modification

The surface wettability of a substrate was modified to be hydrophilic by O_2_ plasma treatment with 200 W, 160 sccm for 5 min using a plasma system (Femto Science, Hwaseong, Korea). Hydrophobic surface modification was done by flowing 100 sccm of C_4_F_8_ gas for 30 min at 160 W, and this procedure was repeated 3 times [36,37]. 

### 2.4. Characterization of Materials

The solidity and porosity of the substrates were calculated by Equations (1) and (2), where m (g) is sample mass; A (cm^2^) is sample area; t (cm) is sample thickness; ρ (g/cm^3^) is polymer density (1.21 g/cm^3^ for PLA; 1.05 g/cm^3^ for PS). Apparent volume and total pore volume were calculated by Equations (3) and (4).

Solidity (unitless) = m/(A × t × ρ)(1)

Porosity (%) = [1 − solidity] × 100 (%)(2)

Apparent volume (mm^3^) = Surface area (mm^2^) × Thickness (mm) (3)

Total pore volume (mm^3^) = Apparent volume (mm^3^) × Porosity (4)

The mean diameter of fibers was obtained by measuring at least twenty fibers from scanning electron microscopy (SEM) images. The substrates used in this study are described in Table 1. The pore size distribution of filter media was measured with a capillary flow porometer (CFP-1500AE, Porous Materials Inc., Ithaca, NY, USA). The morphology of bacteria and substrates were observed by FE-SEM (Supra 55VP, Carl Zeiss, Jena, Germany). Bacteria cells were fixed by osmium tetroxide vapor (2% *w/v*) for 24 h. Before SEM analysis, all samples were sputter-coated with Pt at 20 mA for 180 s (EM ACE200, Leica, Wetzlar, Germany). The sample description and characteristics are summarized in Table 1. The number noted next to fibers is the average fiber diameter.

Static contact angle (CA) was measured using a contact angle analyzer (Theta Lite, KSV Instruments Ltd., Espoo, Finland). The CA of the liquid drop with the volume of 3 μL was measured in 5 s upon deposition on the textile substrates or bacteria dried-plateau. The average value of at least 6 measurements was recorded. Bacteria surface was obtained by culturing the cells on an LB agar plate and air-drying for 3 h. The surface energy of each polymeric and bacteria surface was calculated using the CAs of water and methylene iodide and applying the Owens–Wendt model [38].

### 2.5. Quantification of Surface-Adhered Bacteria

For bacterial binding to a substrate, a film or fibrous sample substrate of 1 cm × 1 cm was immersed in a 1 mL of bacterial culture in LB broth, with the initial OD_600_ of 0.75 and 0.80 that corresponding to ≈4.8 × 10^7^ cells/mL for *E. coli* and *S. aureus*, respectively. Then, 1 mL of each bacterial culture and substrate was placed in a 24-well plate and incubated at 37 °C for 1 h at 100 rpm. After incubation, the weakly adhered bacteria on the substrate were removed by two times of gentle rinsing with 1 mL of PBS at 100 rpm for 5 min each. The remaining bacteria on surfaces were quantified by the INT staining method as follows [39]. The surface-adhered bacteria were stained by treating with 1.2 mL of 1.65 mM INT/PBS solution at 37 °C for 4 h, and the purple-formazan was extracted with 1 mL of DMSO. The optical density of the extracted formazan was measured at 470 nm (OD_470_) by a microplate reader spectrophotometer (SpectraMax 190, Molecular Devices LLC, San Jose, CA, USA). The number of bacteria was quantified by fitting the standard curve between OD_470_ and colony-forming units (CFU).

## 3. Results and Discussion

### 3.1. Effect of Wettability on Cell Adhesion

**Figure 1:**
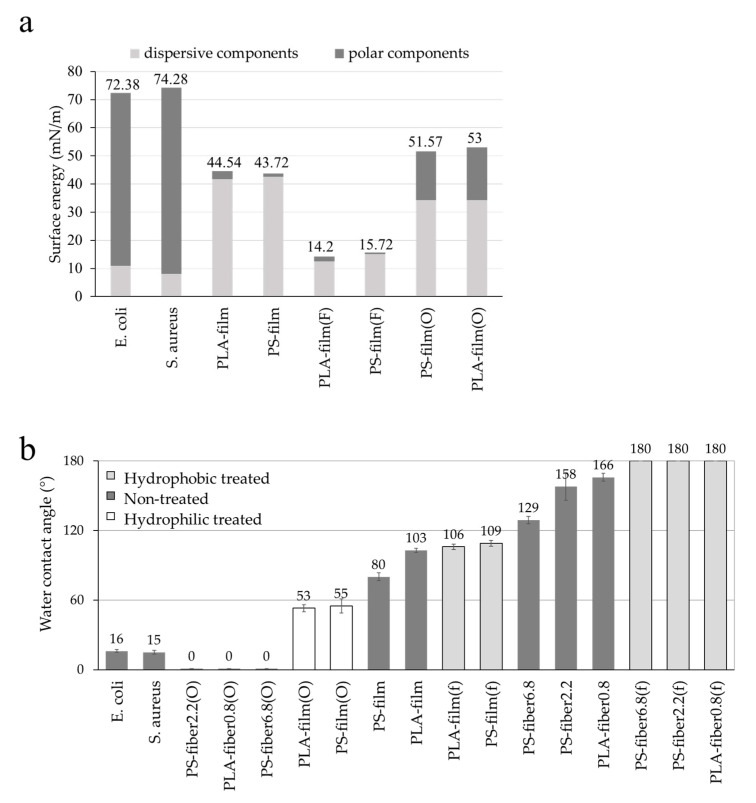
Surface energy and wettability. (**a**) Surface energy with polar and dispersive components; (**b**) water contact angle of different surfaces.

Figure 1 shows the surface energy components and wettability of substrates. The surface energy was estimated by employing the Owens–Wendt model [40]. The Owens–Wendt model assumes that the surface is smooth; thus, the surface energy was calculated from the smooth film surfaces, and then the surface energy of the fibrous surface was regarded as the same. However, the presence of surface roughness of fibrous materials contributed to the wettability, as the Wenzel model [41] and Cassie–Baxter model [42] explain. Particularly, the Cassie–Baxter model [42] explains that the contact angle increases as the solid area fraction, which is in direct contact with the liquid drop, is reduced. From a rough surface where the air is trapped between the surface protrusions, the solid area fraction is less than 1, and this solid area fraction value determines the apparent contact angle, according to the Cassie–Baxter model. On the other hand, the Wenzel model [41] assumes that the droplet fully fills the cavity between the roughened protrusions, and the presence of surface roughness affects the apparent contact angle. Regardless of detailed assumptions, both theories commonly conclude that the existence of roughness intensifies the tendency of wettability of smooth surface. That is, when a smooth surface is hydrophobic with contact angle (CA) > 90°, the roughened surface with the same surface energy further enhances the hydrophobicity; likewise, when roughness is introduced to a hydrophilic surface, it further increases the hydrophilic tendency on the roughed surface [43].

The results of contact angles and surface energy components of all samples are shown in Figure 1. The untreated PLA and PS showed similar overll surface energy, while their dispersive and polar components of surface energies were slightly different due to the difference of chemistry. When the surfaces of PLA and PS were treated by C_4_F_8_ PECVD, the surface energy of both surfaces became very similar to the same level of polar and dispersive components as the same coating was applied. The fluorinated surface showed lowered CAs either for the films or the fibers compared to the untreated surfaces (Figure 1b). Likewise, O_2_ plasma increased the substrate’s polar components and the total surface energy at a similar level for PLA and PS, enhancing the wettability. With hydrophilic surface modification, the CAs of O_2_-treated substrates were lowered compared to the untreated substrates. The results for C_4_F_8_ and O_2_ plasma treatments confirmed the successful modification of surface wettability, changing the surface energy components. The bacteria cells showed hydrophilic property from CA measurement and the estimation of surface energy; thus, they may interact better with hydrophilic surfaces. 

**Figure 2:**
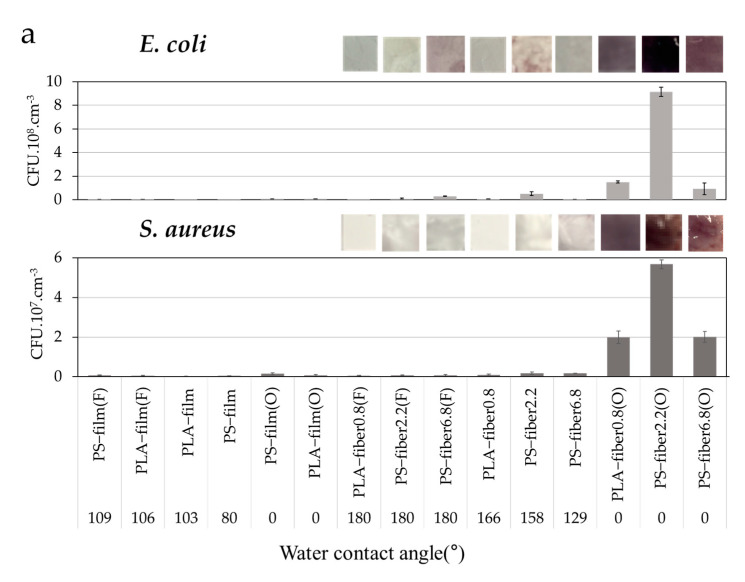

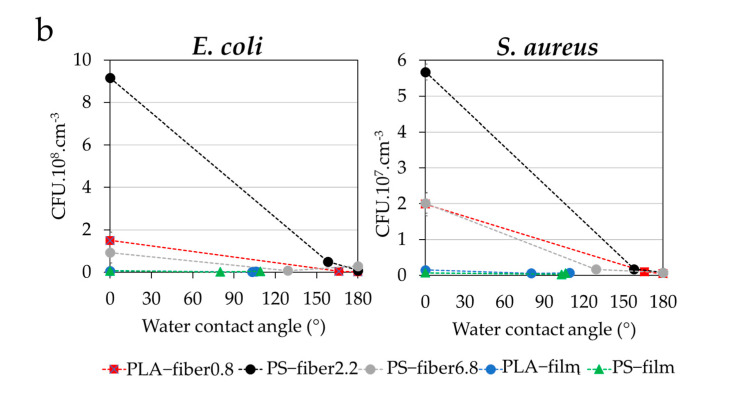
Bacterial adhesion rate under varied substrates. (**a**) Iodonitrotetrazolium chloride (INT) staining of *E. coli* and *S. aureus* adhesion on substrates with varied wetting properties; (**b**) *E. coli* and *S. aureus* adhesion as a function of surface contact angle.

Correspondingly, hydrophilic fiber samples with O_2_ plasma-treated had higher bacteria adhesion compared to the hydrophobic substrates (Figure 2a). The adhesion result was correlated with the wetting properties of material surfaces. The bacteria tested in this research were evaluated to be hydrophilic and preferred to attach to the hydrophilic substrates [44,45]. During the PBS rinsing process, the bacteria that attached to the hydrophobic surfaces were easily removed because of weak interaction with the surfaces. From the results, the wettability of the substrates was a dominant factor affecting the bacteria adhesion, in which hydrophilic surfaces showed higher adhesion. As shown in Figure 2b, the bacterial adhesion decreased as the CA increased for both *E. coli* and *S. aureus*. However, the result was at odds with some previous research [46,47,48], in which *E. coli* with a lipopolysaccharide cell wall is likely to attach better on the hydrophobic surface; but it is still controversial whether *E. coli* would always show favorable adherence on hydrophobic surfaces [49,50,51]. As the bacteria and substrates were incubated in aqueous media, the hydrophilic bacterial medium would favor interacting with the hydrophilic substrates, effectively carrying the cells into the fibrous substrates. A similar result was reported by Bajpai et al. [31], in which hydrophobic fabrics, including polyester, did not provide *E. coli* adhesion. 

The wettability alone cannot explain the significant difference of adhesion between the hydrophilic films and the hydrophilic fibers shown in Figure 2. Thus, the wettability may not the only factor affecting the adhesion, but other factors such as pore structure may also participate in bacteria adhesion. Even though textile’s most distinctive characteristic that might affect bacteria adhesion is the porosity, there have been few studies that explain the effect of pore characteristics on bacteria adhesion. Our study particularly discusses the varied morphological and pore effects on bacterial adhesion, analyzing the porosity percentage, total pore volume, and the pore size distribution in association with the bacteria adhesion, and this makes this paper novel compared to previous research.

**Figure 3:**
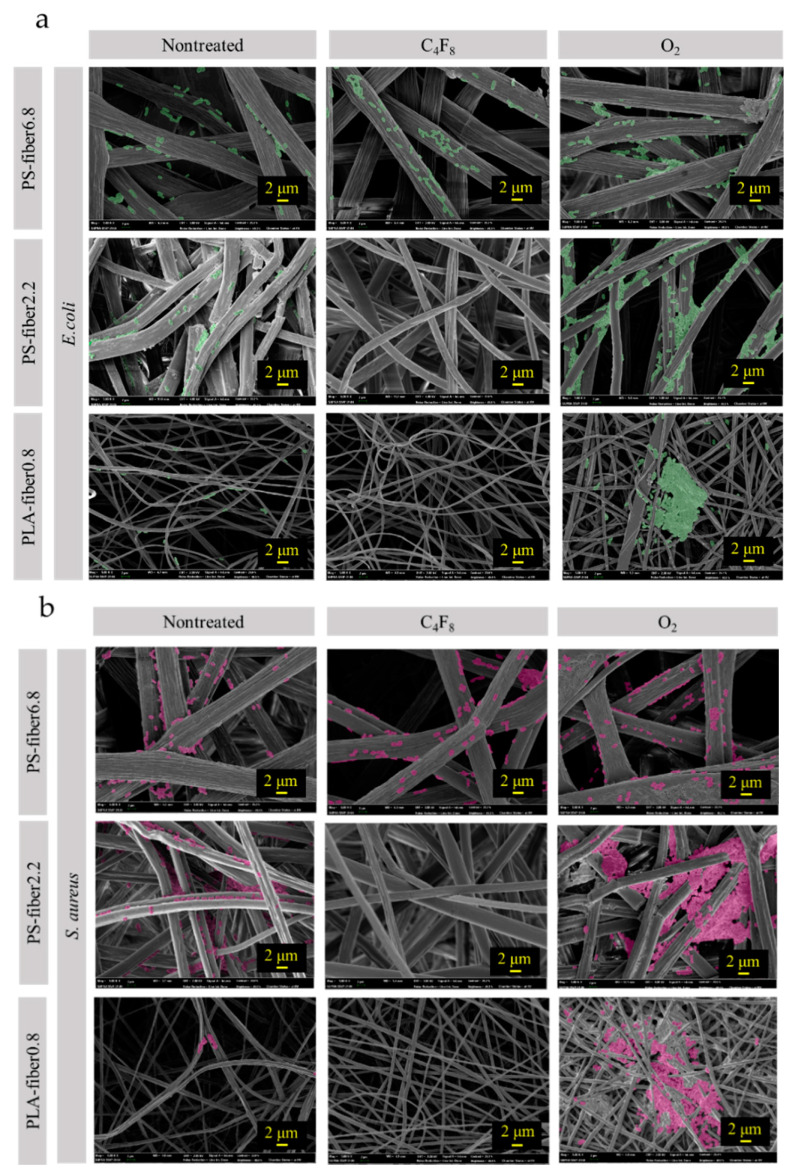
Bacterial adhesion on different substrates. (**a**) *E. coli*; (**b**) *S. aureus*.

SEM images of *E. coli* and *S. aureus* adhered on different fibers are shown in Figure 3. Both *E. coli* and *S. aureus* adhesion had a similar adhesion trend but with a higher adherence for *E. coli*. The rod-shaped *E. coli* would have a larger interactive surface area than the *S. aureus* in a spherical shape, which may have affected the number of adhered cells [52,53,54]. In general, O_2_ plasma-treated hydrophilic substrates showed a high bacteria adhesion, while C_4_F_8_-treated, superhydrophobic substrates showed a very low bacteria adhesion. However, unlike the quantitative measurements shown in Figure 2, SEM images provide only qualitative information on how surface adherence is observed. It does not count the cells adhered inside the pores of the material; thus, the observed adhesion may not accurately represent the total number of adhered bacteria. The effects of pore characteristics and packing density on the adhesion were further investigated in the next section.

### 3.2. Effect of Morphology and Pore Characteristics on Bacteria Adhesion

From Figure 3, the tendency of cell adhesion on different substrates was observed. Bacteria were rarely observed from film surfaces; thus, the images of films were not included in Figure 3. For PS-fiber6.8 with a larger fiber diameter, most bacteria were observed on the fiber surface, and little was observed in the space between the fibers. For PLA-fiber0.8, which had submicron-sized fibers, relatively high amount of cells were observed from the surface. PLS-fiber0.8 with the smallest fibers were rather densely packed than other fibrous webs, and a higher loading of bacteria at the surface was observed, clogging the surface pores of the web. Spatial compactness between fibers seemed to limit bacteria cells’ depth loading, somewhat limiting the intrusion of bacteria into the inner pores. While the extent of surface-adhered bacteria looked similar for PLA-fiber0.8 and PS-fiber2.2, the quantitative measurement of cells from the extracts revealed a higher cell loading for PS-fiber2.2. It can be speculated that more bacteria cells were present inside the pores of the web for PS-fiber2.2 than for PLA-fiber0.8, resulting in higher total adhesion in the volume. In speculating that the pore volume and/or pore size of materials would affect the cell adhesion, the pore characteristics were further investigated.

**Figure 4:**
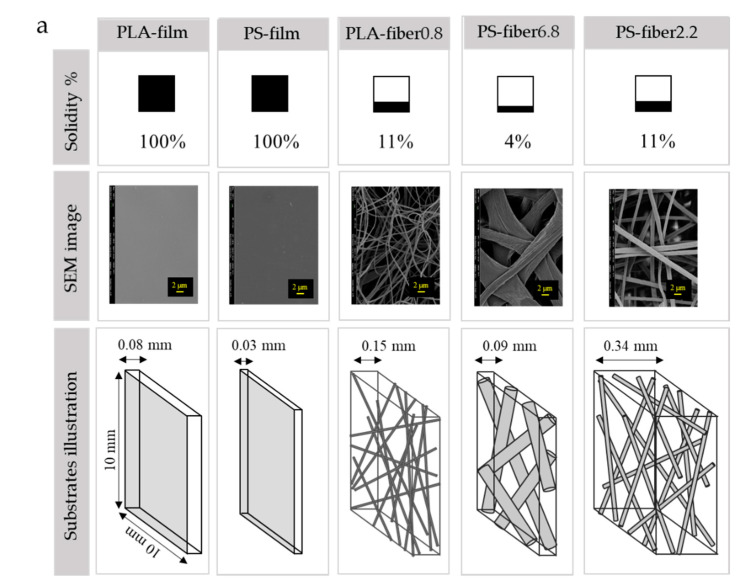

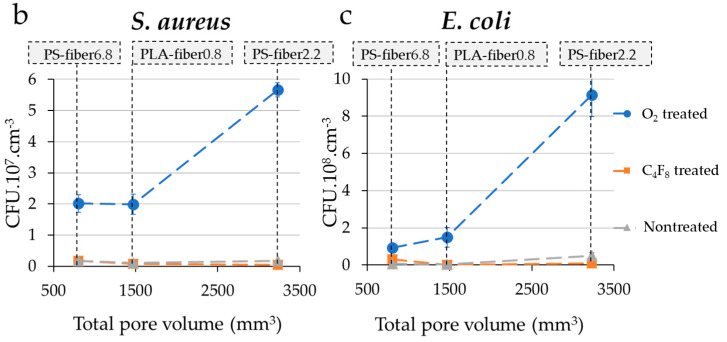
Morphology and pore characteristics of substrates. (**a**) Solidity, SEM image (inserted scale bar is 2 μm), and schematic illustration of the relative size of fibers and pores for different substrates; (**b**) *S. aureus* adhesion with a varied pore volume of substrates; (**c**) *E. coli* adhesion with a varied pore volume of substrates.

In Figure 4, the solidity of the fibrous substrate is illustrated. It was assumed that films do not have inner pores, and the solidity was estimated to be 1 (porosity 0%). Compared to a film substrate, a fibrous web had a considerably small solidity value with a notable value of porosity [55]. The film, which had a smooth surface with zero porosity, showed a lower degree of bacteria adhesion compared to electrospun webs, regardless of surface treatments. It appeared that a smooth surface with little porosity is advantageous for the antifouling property, as the bacterial cells cannot intrude into pores for a firm attachment. Particularly, bacterial attachment on the surface with little surface roughness and negligible pores makes bacteria adhesion unstable, leading to easy detachment when applying the mechanical stress at rinsing. On the contrary, fiber webs with random surface roughness and extreme porosity showed much higher bacteria adhesion (Figure 3b and Figure 4c) [31,32,34]. Similar to the preceding, Bajpai et al. [14] demonstrated that fabrics with a rough surface, such as cotton, had a great bacterial adherence. Puddles and random-sized pores that are accessible from the web surface would allow strong attachment of bacteria. Once bacteria settle inside the pore, they are hardly removed with the external stress with the rinsing procedure. 

Among the O_2_-treated hydrophilic substrates, PS-fiber6.8(O) with the largest porosity (96%) showed a lower adhesion than PS-fiber2.2(O) and PLA-fiber0.8(O). Thus, factors other than porosity percentage may also play a role in adhesion. Between PLA-fiber0.8 and PS-fiber2.2, the basis weight is a little higher for PLA-fiber0.8, but the thickness is considerably higher for PS-fiber2.2 (Table 1). The solidities (or porosity percentages) of PLA-fiber0.8 and PS-fiber2.2 were the same. The results showed that while the porosity (%) was the same for PLA-fiber0.8 and PS-fiber2.2, PS-fiber2.2 had a higher adhesion. Based on the results, it was thought that the absolute pore volume, rather than porosity, may be another important factor influencing the bacteria adhesion. In this study, the apparent volume and the total pore volume were calculated by Equations (3) and (4), and the total pore volume was analyzed as an important factor affecting the bacteria adhesion.

The porosity percentage, apparent substrate volume, and the web’s total pore volume are disproportionally related. According to Figure 4, PS-fiber2.2 had the highest total volume and lowest solidity, making it a much fluffier fiber sample with the highest total pore volume. A large volume of total pores with hydrophilic nature allowed significantly higher bacteria adhesion, where the inner pores acted as trap sites for penetrated bacteria inside the substrates. Once bacteria were trapped inside the sample, it was difficult to detach the cells from the web. Figure 4 demonstrates that the higher pore volume of PS-fiber2.2(O) affected the higher amount of adhered bacteria on the web. Compared to PS-fiber2.2(O), PLA-fiber0.8(O) showed a lower pore volume with a compact structure, resulting in a lower adhesion amount. As a result of the denser structure of PLA-fiber0.8(O), the penetration of bacteria into the web seemed to be limited, lowering the adhesion.

Similarly, Karger et al. [56] demonstrated that bacteria preferred to adhere to the gap between the fibers, and this result was following our finding. In our study, the gap between the fibers was smallest for PLA-fiber0.8, and this led to the smallest total volume; the combined effect of lower pore volume and the smaller fiber-to-fiber gap led to a lower bacteria adhesion. It is noted that this relationship was explained for hydrophilic surfaces, which showed a relatively higher extent of bacteria adhesion. As the wettability was consistent for those O_2_-treated substrates, the distinctive factors among those samples were morphology and pore characteristics, and it allowed the analysis of pore effects.

**Figure 5:**
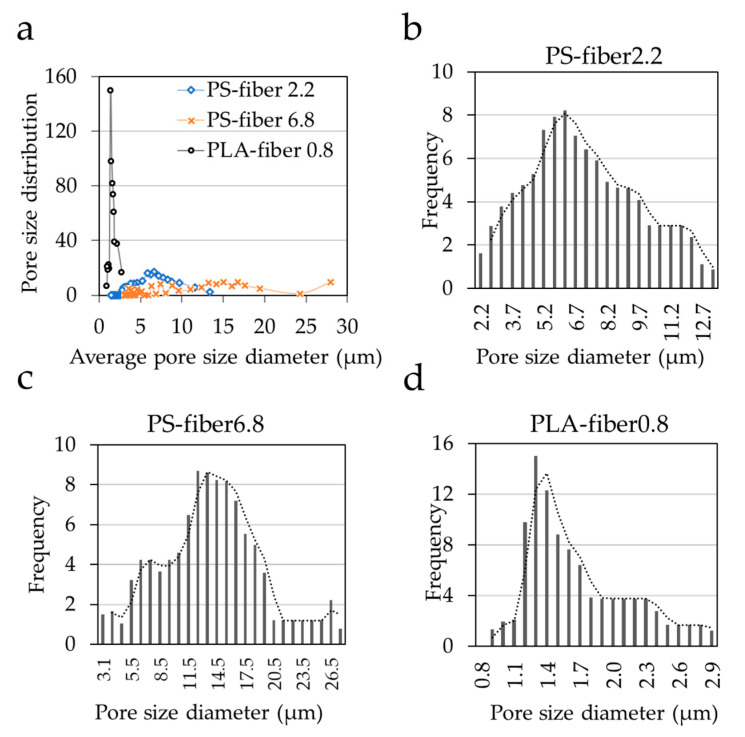
Pore size distribution (**a**) pore size distribution vs. average pore size diameter in μm of all electrospun webs; (**b**–**d**) Electrospun webs pore size distribution vs. diameter in μm of PS-fiber2.2, PS-fiber6.8, and PLA-fiber0.8.

To further examine the effect of pore characteristics on the bacteria adhesion, pore size distribution of the web for PS-fiber2.2, PS-fiber6.8, and PLA-fiber0.8 was measured (Figure 5). PLA-fiber0.8 showed a narrower size distribution with smaller pores, while PS-fiber6.8 depicted a wider size distribution with larger pores. Although PLA-fiber0.8 and PS-fiber6.8 had similar porosity (89%), they represented a considerable difference in pore size distribution. While the total pore volume itself seemed to be an influential factor for the bacteria adhesion, the relative size of pores to the cell appeared to be another factor determining the cell adhesion and retention. When the pore size is too large, bacteria would easily intrude in the web pores, but they would also easily escape. If the pore size is too small or not large enough to endure bacteria, bacteria cannot properly intrude the pores but bump into the surface, leading to the easy isolation from the substrates. For such reasons, the PS-fiber2.2(O), which showed pores of 2-–12 μm with a high pore volume, showed a higher bacterial adhesion than the substrates with similar wettability. PS-fiber6.8 had much larger pores of 3–26 μm, which would allow easier de-trapping of adhered bacteria. From the SEM images in Figure 3, bacteria adhered the most on the surface of fibers, and they were not observed in between the fibers, which was probably because they were removed during the rinsing process. 

With the growing concerns about the spreading of infectious diseases by microbial, it is imperative to control the bacteria adhesion and growth on textiles. This study is concerned with textile parameters and design insights to control the bacteria adhesion in the liquid medium. Textiles as porous materials, the pore volume, and the pore size distribution of the material need to be included as important design parameters. The results indicated that wettability, packing density, pore size, and volume were involved with cell adherence and retention in the material. The results of this study can be applied in designing disposable hygiene textiles or protective equipment made of nonwovens. For example, a superhydrophobic nonwoven web with reduced pore volume and close packing would be advantageous in circumventing the adhesion. However, it is still challenging to predict the adhesion with the time factor. Further research is needed on the long-term biofouling, such as biofilm formation, to disclose the ambiguity of the adhesion mechanism as a function of time.

## 4. Conclusions

This work sought to determine the effect of wettability and pore characteristics of textiles on the bacterial adhesion in the liquid medium. The textile parameters including wettability, surface energy, porosity percentage, pore volume, and pore size distributions were analyzed in association with bacteria adhesion. Substrates with different levels of surface energy and varied pore characteristics were employed to identify the critical factors influencing cell adhesion. The surface energy of the material was modified via a plasma process employing O_2_ gas for hydrophilic treatment or C_4_F_8_ for hydrophobic treatment. Based on the result, the substrate’s wettability was the primary factor influencing the cell adhesion, where higher adherence was observed from hydrophilic substrates. The fibrous morphology not only provided more surface area for bacteria adhesion but also affected the wettability. The surface roughness of the fibrous web further enhanced the wettability of a high surface energy material and promoted the cell adhesion. The pore volume and pore size, rather than the porosity itself, were other important factors affecting the bacteria adherence and retention. The high packing density with small pores prevented depth loading of bacteria cells, reducing the bacteria retention of the material. Thus, antifouling hygiene textiles can be designed with superhydrophobic materials with reduced pore volume and small pores.

This study is designed to meet the high demand for anti-fouling textiles. It is novel that pore characteristics, which are unique to textile materials, were considered in interpreting the interaction between bacteria cells and fibrous materials. By understanding the material parameters that affect the bacteria adhesion, the proper design of textiles could be suggested to decrease the adherence of infectious bacteria. The findings of this study would contribute to developing anti-biofouling textiles with potential applications to hygiene products and protective garments that prevent microbial infection. Further research is necessary that accounts for the time factor on the adhesion to reveal the long-term biofouling and biofilm formation.

## Figures and Tables

**Table 1:** Substrate sample description and characteristics.

Code	Description	Thickness (mm)	Basis Weight (g/m^2^)	Solidity (Unitless)	Porosity (%)	Mean Fiber Diameter (μm)	Volume (mm^3^)	Total Pore Volume (mm^3^)
PLA-film	PLA film	0.08 (±0.01)	72.6 (±7.7)	1.00	0	N/A	8	0
PLA-film(O)	O_2_ plasma-treated							
PLA-film(F)	C_4_F_8_ plasma-treated							
PLA-fiber0.8	electrospun PLA fiber	0.15 (±0.02)	19.6 (±4.0)	0.11	89	0.8 (±0.1)	15	1470
PLA-fiber 0.8(O)	O_2_ plasma-treated							
PLA-fiber 0.8(F)	C_4_F_8_ plasma-treated							
PS-film	PS film	0.03 (±0.01)	33.4 (±2.1)	1.00	0	N/A	13	0
PS-film(O)	O_2_ plasma-treated							
PS-film(F)	C_4_F_8_ plasma-treated							
PS-fiber6.8	Electrospun PS fiber	0.09 (±0.00)	11.0 (±1.4)	0.11	89	6.8 (±1.9)	9	801
PS-fiber6.8(O)	O_2_ plasma-treated							
PS-fiber6.8(F)	C_4_F_8_ plasma-treated							
PS-fiber2.2	Electrospun PS fiber	0.34 (±0.05)	17.0 (±5.5)	0.04	95	2.2 (±0.3)	34	3230
PS-fiber2.2(O)	O_2_ plasma-treated							
PS-fiber2.2(F)	C_4_F_8_ plasma-treated							

## Data Availability

The data presented in this study are available on request from the corresponding author.
